# Soil Aggregate Stability and Grassland Productivity Associations in a Northern Mixed-Grass Prairie

**DOI:** 10.1371/journal.pone.0160262

**Published:** 2016-07-28

**Authors:** Kurt O. Reinhart, Lance T. Vermeire

**Affiliations:** United States Department of Agriculture-Agricultural Research Service, Fort Keogh Livestock & Range Research Laboratory, Miles City, Montana, United States of America; US Geological Survey, UNITED STATES

## Abstract

Soil aggregate stability data are often predicted to be positively associated with measures of plant productivity, rangeland health, and ecosystem functioning. Here we revisit the hypothesis that soil aggregate stability is positively associated with plant productivity. We measured local (plot-to-plot) variation in grassland community composition, plant (aboveground) biomass, root biomass, % water-stable soil aggregates, and topography. After accounting for spatial autocorrelation, we observed a negative association between % water-stable soil aggregates (0.25–1 and 1–2 mm size classes of macroaggregates) and dominant graminoid biomass, and negative associations between the % water-stable aggregates and the root biomass of a dominant sedge (*Carex filifolia*). However, variation in total root biomass (0–10 or 0–30 cm depths) was either negatively or not appreciably associated with soil aggregate stabilities. Overall, regression slope coefficients were consistently negative thereby indicating the general absence of a positive association between measures of plant productivity and soil aggregate stability for the study area. The predicted positive association between factors was likely confounded by variation in plant species composition. Specifically, sampling spanned a local gradient in plant community composition which was likely driven by niche partitioning along a subtle gradient in elevation. Our results suggest an apparent trade-off between some measures of plant biomass production and soil aggregate stability, both known to affect the land’s capacity to resist erosion. These findings further highlight the uncertainty of plant biomass-soil stability associations.

## Introduction

Development of stable soil aggregates is thought to depend on a combination of plant roots and mycorrhizal fungi [1–5]. Several studies report a positive association between plant biomass and soil aggregate stability [4, 6]. Plant cover [7–9] and plant roots [3, 4, 10] have also been positively correlated with soil aggregate stability. Additionally, soil aggregate stability is widely interpreted as an indicator of rangeland health in the USA [11–13] and may even function as an important ecosystem property [14].

In contrast, soil aggregate stability was not positively associated with crop yield [15], plant cover [8, 16, 17], total peak aboveground biomass [18], or root biomass [19, 20]. Furthermore, rangeland health assessments in Canada do not include soil aggregate stability [21]. Some soil physics experts also contend that simplified measures of soil structure (e.g. aggregate stability) do not reliably indicate important soil processes [22]. Thus, there is varying evidence and perspectives on the importance of soil aggregate stability to plant biomass, ecosystem functioning, and rangeland health [18, 22].

Other factors that influence plant productivity, such as plant available nutrients, plant species composition, soil moisture, and water infiltration [6, 17, 18, 23], may affect the association between plant biomass and soil aggregate stability. To help reconcile the importance of soil aggregate stability as an indicator of plant biomass in the Northern Great Plains, we tested whether measures of soil aggregate stability explained variation in multiple measures of plant biomass. We quantified local associational patterns (i.e. mensurative experiment) to address the following questions: (1) Is grassland biomass (dominant graminoids and roots) positively associated with soil aggregate stability? and (2) Are grassland productivity-soil aggregate stability associations impacted by plant community composition (i.e. are individual plant species associated with divergent soil aggregate stabilities)? We predicted water-stable aggregates would be a useful indicator of peak aboveground and root biomasses. Local (e.g. plot-to-plot) variation in plant and soil properties was used to uncover locally applicable indicators of plant biomass [24].

## Methods

### Study system and site

Research was conducted on a loamy ecological site at the USDA-Agricultural Research Service’s Fort Keogh Livestock and Range Research Laboratory (Fort Keogh, 22,000 ha), Montana, USA. No special permits were required as the researchers were fully authorized to conduct research at the research station. Fort Keogh is centrally located in the Northern Great Plains Steppe ecoregion which spans five states in the USA and two Canadian provinces [25]. Mean annual precipitation was 34 cm (1937–2011) with most occurring during May and June. Above-ground annual productivity for this system peaks from June to July and is dominated by perennial C_3_ graminoids [26].

The study site (46°18’20.8”N, 105°58’42.8”W) is an upland plain with a gentle slope (1.05° slope) and fine loamy soil (Eapa loam, frigid Aridic Argiustolls). The site’s vegetation is representative of a common calcareous grassland type in the Northern Great Plains [25, 27] and is seemingly phosphorus limited [23]. Three graminoid species (*Carex filifolia*, *Hesperostipa comata*, and *Pascopyrum smithii*) make up 73% of the total peak plant biomass.

**Fig 1:**
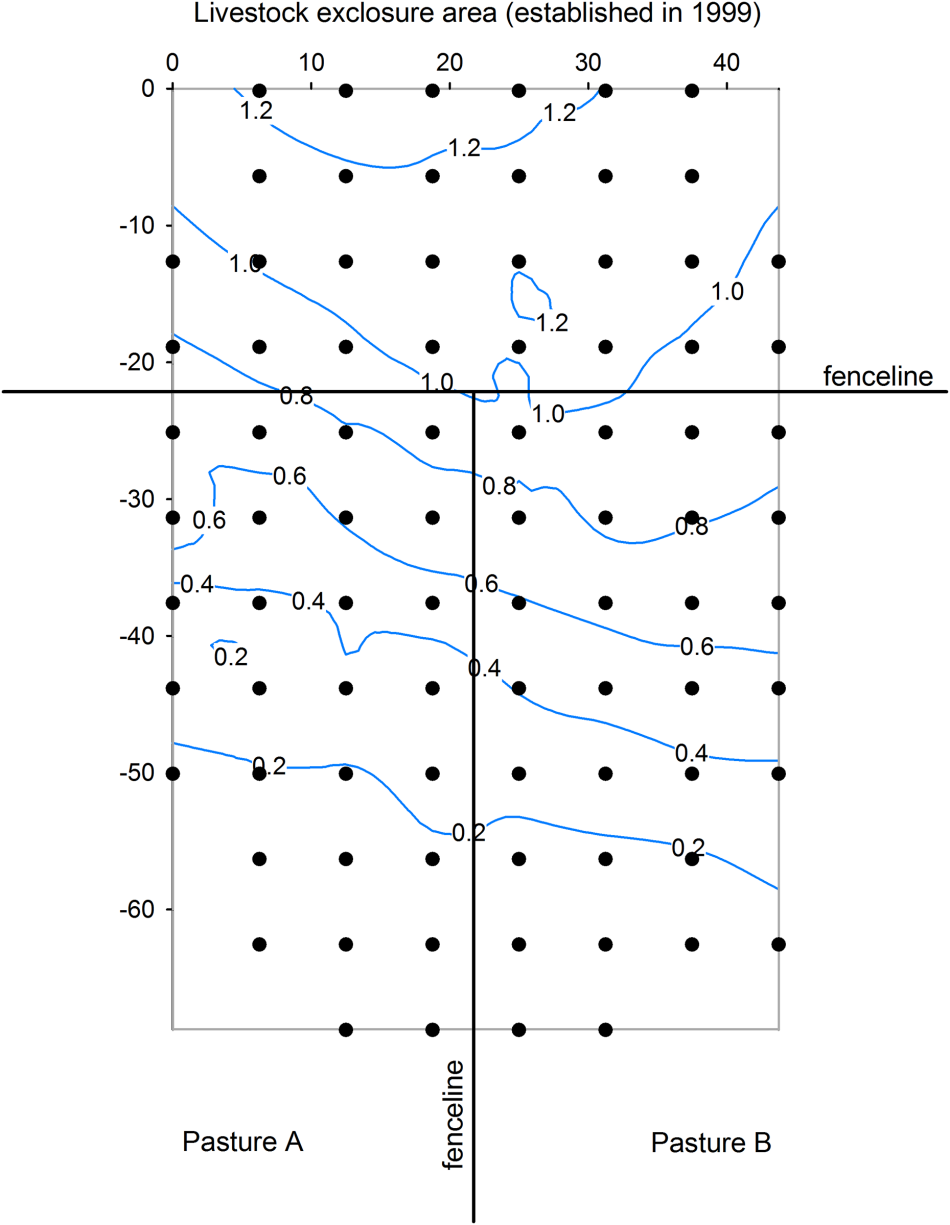
Map of the sampling area (0.3 ha). Sampling was divided equally among three adjacent areas: a livestock exclosure and two adjacent pastures (A, B) grazed annually by cows. Individual sampling points are shown (●), axes’ units are meters, and contour lines represent elevation gain (m) relative to the lowest point in the sampled area.

The study was conducted in a 0.3 ha area. The small size of the study area helped to control for many abiotic factors (e.g. soils, weather/climate), but still included local variation in plant productivity and other factors. Nearly one third (0.1 ha) of the sampled area was within a livestock exclosure established in 1999 (Fig 1). The other two thirds were equally divided among two grazed pastures. Elevation gain (m) relative to the lowest sampled point was determined for 111 points, including all 84 sampled points, with elevation measurements from a leveling system (model 650, Spectra-Physics Laserplane, Lexington, KY). Additional site description details are described by Reinhart et al. [18].

Rangeland ecologists routinely compare data collected from two or more pastures [13]. Such comparisons are complicated by the fact that a pasture may contain several ecological sites (i.e. related soil series), soil series, and plant communities. A common approach is to standardize comparisons among pastures by either ecological site or soil series [11, 28]. Thus, all of our data were collected from a single soil series. Our study does not control for additional possible sources of variation (e.g. soil texture) which is often important when comparisons span regional abiotic gradients [29]. We assumed that for soil aggregate stability to be a useful indicator of plant biomass (i.e. reliably predict local variation in plant biomass) that its predictive power must be independent of additional factors.

### Plant biomass and composition

We determined peak annual aboveground biomass and plant community composition for 0.25-m^2^ quadrats July 5–12, 2011. Quadrats were positioned 0.5 m to the east of 84 fixed points (Fig 1). The clipped vegetation was separated by dominant species and functional groups. Dominant species included four graminoids (*Carex filifolia*, *Hesperostipa comata*, *Koeleria macrantha*, and *Pascopyrum smithii*) and one cactus (*Opuntia polyacantha*). Functional groups included: forbs, other grasses, and shrubs (*Artemisia* spp.). Plant material was dried to a constant weight, separated by current-year and older material, and weighed. Because *Opuntia* is difficult to dry, a correction calculation (0.2 × fresh weight), derived by researchers at Fort Keogh, was used to calculate the dry weight of *Opuntia*. Unless stated, analyses are of total dominant graminoid biomass which represent the summed biomass for *Carex filifolia*, *Hesperostipa comata*, and *Pascopyrum smithii* (73% of total peak annual biomass).

### Root biomass

Soil was cored with an 87 mm diameter soil column cylinder auger (Sunvalley Solutions Inc./Eijkelkamp, FL, USA), which was hammered into the soil using a hand-held jackhammer powered with compressed air on June 12–17, 2012 immediately (ca. 20cm) west of fixed points (Fig 1). Surface vegetation was clipped prior to coring. Compaction of the soil column during the sampling procedure was corrected based on the measured length of the soil core and the burrow depth (sensu [30]). Compaction was on average 1.1% ± 1.2 standard deviation. The soil cores were segregated into 0–10 and 10–30 cm increments and placed in sealed plastic bags. Soil was cored at multiple increments and sieved to enable collection of complementary data to address related research topics and was not essential for quantifying root biomass. The 77 cores resulted in a total of 154 samples that were brought back to the lab and frozen (-20°C) until processed (ca. 0.14 m^-3^ of field soil). [Field crew availability prevented us from coring all 84 points used to sample vegetation and soil stability.]

Because gathering fine root fragments is tedious, we used a subsampling routine and available sieving equipment to expedite quantification of root biomass. Roots were extracted from three sieve size classes of material (>9.5, 9.5–2, and <2 mm). First, the samples were sieved (9.5 mm sieve) on a mechanical sieve shaker (RO-TAP, RX-29, W.S. Tyler, Mentor, Ohio). Samples were shaken for 1 minute (278 oscillations × min^-1^, 150 taps × min^-1^) and roots were removed by hand from the 9.5 mm sieve. Then the remaining soil from the collection pan was sieved again using a 2 mm sieve. The material (roots and soil) in the sieve and pan were weighed (wet weights) as follows. From the sieve (9.5–2 mm material) and pan (<2 mm material), we removed 10 g subsamples and removed roots from each subsample by hand. Each sample yielded roots from three sieved size classes of material. Roots were dried to constant mass (105°C for 1 day), weighed, combusted at 450°C for 4 h, cooled to 60°C, and reweighed to determine the percentage of ash-free dry mass (AFDM). We determined total root biomass for the >9.5 mm portion as total root biomass>_9.5mm_ = root dry mass × percentage of AFDM. The total root biomass of the two other sieved portions was determined with the equation:

$$total\;root\;dry\;mass_{i}=\frac{root\;dry\;mass_{i}-ash\;mass_{i}}{subsample\;mass_{i}}\times sample\;mass_{i}$$ (1)

where total root dry mass for each material size class (9.5–2, <2 mm) *i*; subsample mass (wet weight≈10 g) for each size class *i*; and sample mass (wet weight) for each size class *i*. For each core sample, we summed the total root dry masses for the three material size classes. We then determined the total root biomass per sampling location by summing the root data for the two soil core depth increments (0–10, 10–30 cm).

We also estimated the abundance of roots of a dominant graminoid species (*Carex filifolia*). We used *C*. *filifolia* because we knew *a priori* that its roots were visually distinct from other species at the site [31]—its roots are coarse and highly pigmented [32, 33]. We visually estimated root biomass of *C*. *filifolia*—like field methods (e.g. Domin and Braun-Blanquet) used to estimate plant cover. Specifically, we examined all the samples and selected 10 spanning a gradient in root dry weights of *C*. *filifolia*. We then photographed each reference sample which contained a mix of root fragments and soil aggregates >9.5 mm. [Since we destructively measured roots from soil cores, we used material (soil and roots >9.5 mm) from the soil aggregate stability tests (see next subsection) to estimate *C*. *filifolia* root biomass.] Coarse and highly pigmented roots were separated from the soil by hand, dried to constant weight, and weighed indicating a gradient in root biomass (0.10, 0.18, 0.34, 0.37, 0.66, 1.00, 2.06, and 5.34 g per sample). The root biomass of *C*. *filifolia* was then estimated for all other samples by visually matching the amount of coarse pigmented roots in each sample to the most similar reference sample photograph.

### Soil aggregate stability

We measured % water-stable (soil) aggregates following standard methods [34] described in detail by Reinhart et al. [18]. At the location where soil was to be collected, we clipped plant material and removed detritus. We carefully extracted a larger than needed soil sample using a narrow bladed (13 cm) shovel on May 8, 2012 ca. 0.5 m to the south of 84 sampling points (Fig 1). The soil sample was then cut down to 10 × 7 × 6 cm (10 cm length = 0 to -10 cm soil depth). This resulted in a cuboid soil sample free of soil compaction from the extraction process which was placed in a sealed plastic bag. The soil samples were transported in stackable plastic boxes and brought back to the lab. In the lab, soil samples were lightly massaged to break up the soil along natural breakpoints [35]. The samples, representing 0–10 cm of soil, were air dried to constant weight and stored in plastic bags in stackable boxes until dry-sieved.

The samples were dry-sieved on a mechanical sieve shaker. Each aggregate size class was collected individually from largest to smallest (4–2, 2–1, and 1–0.25 mm). Samples were shaken for 1 minute (278 oscillations × min^-1^, 150 taps × min^-1^). Individual samples representing separate aggregate size classes per sample were weighed and stored in plastic bags in stackable plastic boxes until wet-sieved.

The percentage of water-stable aggregates was determined for three macroaggregate size classes (0.25–1, 1–2, and 2–4 mm) following the operators manual for a wet-sieving apparatus (Sunvalley Solutions Inc./Eijkelkamp, FL, USA). We measured the stability of three separate aggregate size classes to help ensure detection of positive plant-aggregate associations. Aggregates from the dry sieving portions (1, 4, and 4 g, respectively) were placed onto mesh screens a quarter the size of the smallest aggregates (0.0625, 0.25, and 0.5 mm, respectively). Four subsamples per size class were used to estimate % water-stable aggregates per sample. Subsamples were rewetted by capillary action for 10 minutes. Subsamples were wet-sieved for 8 minutes on a mechanical wet-sieving apparatus (stroke = 1.3 cm, at 34 strokes × min^-1^). After wet-sieving, the material collected in the cans was washed gently into weigh boats. Cans were then re-filled with a dispersing agent (0.2 g NaOH), and the coarse material persisting on the sieve was wet-sieved again. At the end of this wet-sieving, we manually broke up any remaining coarse material. The sample was then wet-sieved for an additional two minutes in the dispersing agent solution. The material in the can containing the dispersing solution was then washed gently into another set of weigh boats. The weigh boats were then dried in a convection oven at 110°C, until the water had evaporated, and weighed. The formula for determining the percent water-stable aggregates (corrected for coarse material) was:

$$WSA_{i}=\;\frac{W_{2}-0.2}{W_{1}+(W_{2}-0.2)}\;\times 100$$ (2)

Where *WSA*_*i*_ = water − stable aggregation for each size class *i; W*_1_ = weight of material collected in the cans after the first wet sieving in water in size class *i*; *W*_2_ = weight of material collected in the cans after the second wet sieving in the dispersing agent (weighing 0.2 g) in size class *i*. Analyses were of the mean of subsamples per sample.

### Analysis

The primary purpose of this study was to quantify plant productivity and soil aggregate stability associations (i.e. mensurative experiment). Prior to regression analyses, outliers were identified and removed based on the maximum normal residual method [36] and P< 0.05. (Because of the large number of zeros in the *Carex filifolia* root biomass data, outliers were not removed from those data.) Recall that our aim was not to test livestock management treatments which were not replicated (Fig 1). However, we provide summary statistics (mean and 95% CI) for the three sampled areas (Table 1).

**Table 1:** Mean (±95% CI) plant properties and soil aggregate stability values for three adjacent areas on a relatively flat upland site (0.3 ha) of northern mixed-grass prairie vegetation. Variation among the three areas can be interpreted by whether 95% CI overlap.

site properties	depth (cm)	Area1 (exclosure)	Area2 (pasture A)	Area3 (pasture B)
grass	—	169.4±14.3	137.9±19.3	121.3±17.7
roots	0–30	1,338.5±239.2	589.5±83.6	920.5±184.0
lgWSA	0–10	45.1±4.4	58.5±4.1	55.0±4.1
medWSA	0–10	41.8±3.6	57.1±3.6	49.7±3.3
smWSA	0–10	67.9±2.7	84.5±3.7	75.1±4.4

grass = aboveground peak annual biomass (g × m^-2^) of three dominant graminoid species, roots = biomass of living and dead roots (g × m^-2^; 0–30 cm), lgWSA = % water-stable aggregates (WSA, 2–4 mm), medWSA = % WSA (1–2 mm), and smWSA = % WSA (0.25–1 mm).

Our study’s aim was to identify potentially meaningful associations between plant and soil properties. However, the data were often spatially correlated and violated the assumption of sample independence (i.e. independent observations) for regression analyses i.e. errors were correlated across samples [37]. To address this, we first tested for the presence of spatial autocorrelations with Moran’s I tests using the “ape” package [38]. We measured spatial autocorrelation using an inverse distance weighted residual error matrix. We also visually assessed spatial autocorrelation with spatial variograms. If spatial autocorrelation was present then we conducted regression analyses, with soil aggregate stability as the predictor variable and biomass as the response variable, using generalized least squares (GLS) which account for spatial autocorrelation in model residuals [39, 40]. For the GLS models, we tested six ways of fitting a parametric correlation function (uncorrelated errors, exponential, Gaussian, linear, rational quadratic, spherical) to the residual co-variance matrix which were fit by maximizing the restricted log-likelihood (REML). We selected the model (i.e. uncorrelated errors, exponential, or rational quadratic) with the lowest Akaike information criterion (AIC) score for each predictor variable. After visually checking for normality and homogeneity of residual variances, *C*. *filifolia* root biomass data were log transformed. All tests were performed in R [41], and GLS was conducted with the “nlme” package. Results of simple linear regressions (ordinary least squares [OLS]) are also presented. Qualitative differences in slope coefficients for models based on OLS vs. GLS, e.g. positive association detected with OLS but no appreciable association with GLS, suggest soil aggregate stability may not be a useful indicator of plant productivity.

Plant community dissimilarity was visualized with a non-metric multidimensional scaling (NMDS) ordination with a Bray-Curtis distance matrix using the function “metaMDS” in the “vegan” package [42]. Environmental vectors were fitted to the ordination using the “envfit” function of the “vegan” package. Each vector (environmental variable) was independently fitted to the underlying ordination. The fit (R^2^) of each vector to the ordination using the “envfit” function was assessed with a Monte-Carlo analysis of 1,000 permutations. The environmental vectors dataset included: *Carex filifolia* root biomass, dominant grass biomass, elevation gain, total root biomass, and % water-stable aggregates (0.25–1, 1–2, and 2–4 mm).

## Results

Descriptive comparisons (95% CI) of the three sampled areas (ungrazed, pasture A, and pasture B) indicate some differences in peak annual (dominant) graminoid biomass, root biomass, and % water-stable aggregates (Table 1). Specifically, the grazing exclosure portion had greater dominant graminoid biomass than pasture B and greater root biomass than pasture A. Pasture B also had greater root biomass than pasture A. Our root biomass values (Table 1) were generally less than values reported for another grassland site in the Northern Great Plains (mean total root biomass_0-30 cm depth_ = 1,684 g × m^-2^) [43] but were similar to values from another site at the field station [44]. The grazing exclosure had the lowest levels of % water-stable aggregates. The % water-stable aggregates (0.25–1 mm) was greatest in pasture A followed by pasture B, and the exclosure area. Descriptive differences of separate areas should not be interpreted as effects of treatments on measured properties because results are not for a replicated field experiment (i.e. n = 1 grazing exclosure).

**Fig 2:**
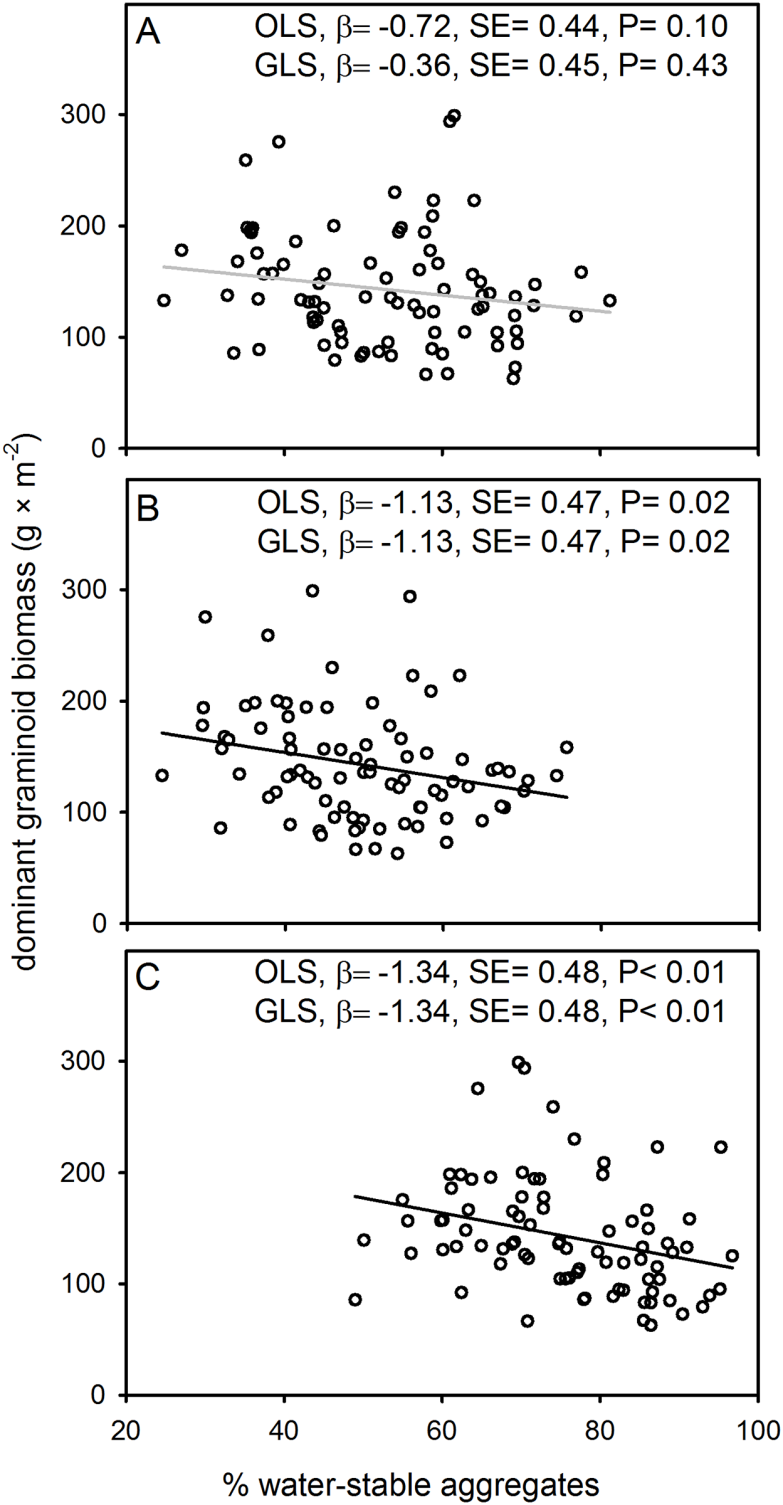
Relationships between % water-stable aggregates and peak annual dominant graminoid (aboveground) biomass (A-C). Panels represent three size classes of macroaggregates: 2–4 mm (A), 1–2 mm (B), and 0.25–1 mm (C) (0–10 cm soil depth). Regression slope coefficients (β), standard errors (SE), and p-values are provided for regressions based on ordinary least squares (OLS) and generalized least squares (GLS). GLS controls for spatial autocorrelation. Best-fit lines (based on OLS) are black when GLS regressions were significant (P≤ 0.05) or gray when nonsignificant (P> 0.05).

Overall, plant and soil properties exhibited varying degrees of spatial autocorrelation (Moran’s I, P< 0.05) indicating that results for simple linear regressions, based on ordinary least squares (OLS), may have increased Type I statistical errors (i.e. false positives). To account for spatial autocorrelation, regressions were repeated with generalized least squares (GLS). Results based on OLS tended to show negative associations between water-stable aggregates and measures of plant biomass while results based on GLS found a mixture of no appreciable associations, marginally significant (0.05 < P ≤ 0.10), and significant (α = 0.05) negative associations between water-stable aggregates and plant properties. Results based on OLS and GLS for dominant graminoid aboveground biomass were similar (Fig 2). Specifically, two size classes (0.25–1 and 1–2 mm) of % water-stable aggregates were negatively correlated with dominant graminoid (aboveground) biomass (Fig 2B and 2C). Additionally, results for GLS indicated a negative association between the largest aggregate size class and total peak annual aboveground biomass (β = -1.10, SE = 0.48, P = 0.02), a marginally significant negative association for the medium sized aggregates (β = -1.01, SE = 0.54, P = 0.06), and no appreciable association for the smallest aggregate size class (β = -0.31, SE = 0.57, P = 0.59; results not shown).

**Fig 3:**
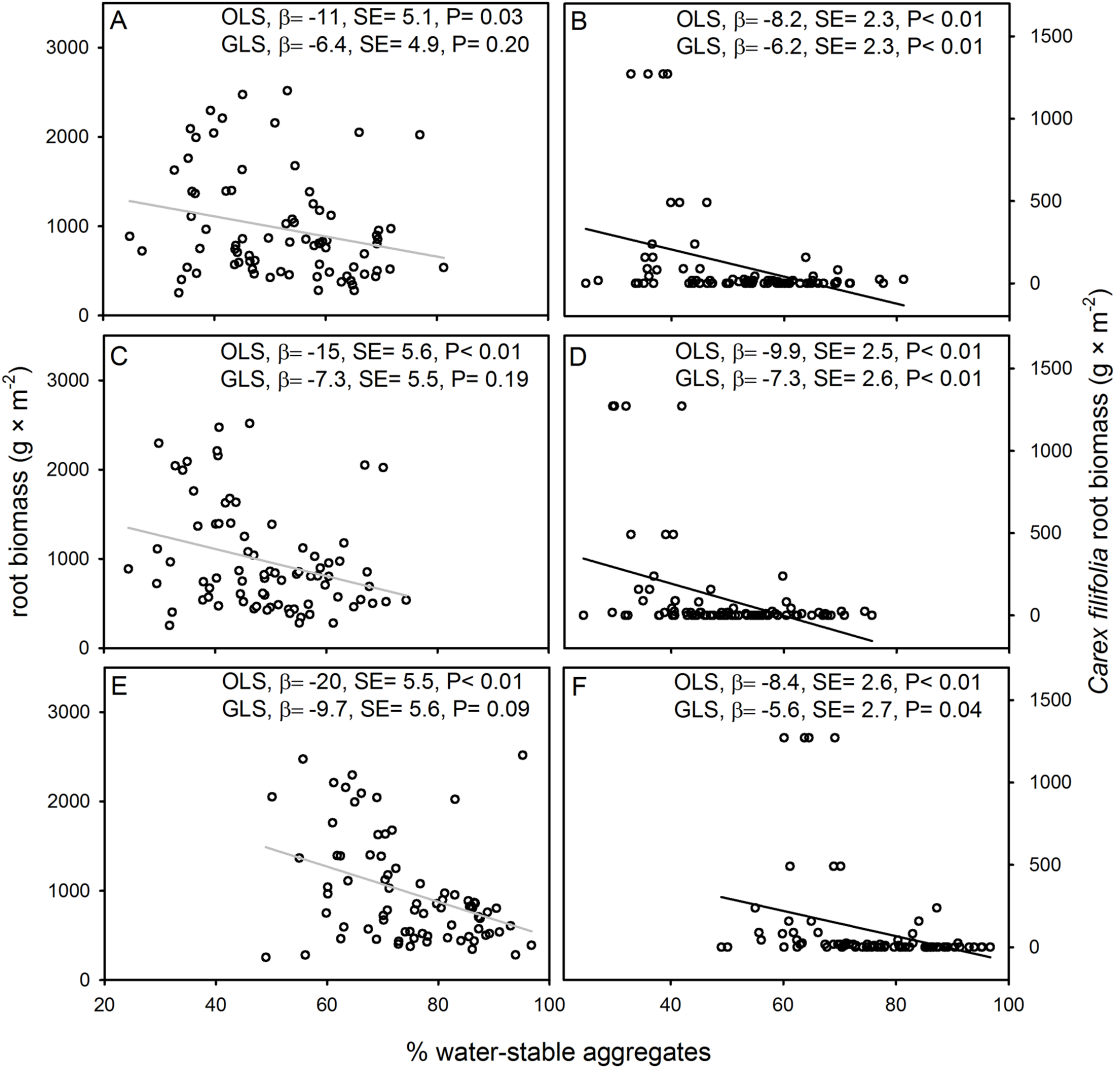
Relationships between % water-stable aggregates and root biomass. Panels show data for either root biomass (A, C, E) (0–30 cm soil depth) or *Carex filifolia* root biomass (B, D, F) (0–10 cm soil depth). Panel rows distinguish data for three size classes of macroaggregates: 2–4 mm (A,B), 1–2 mm (C,D), and 0.25–1 mm (E,F) Regression slope coefficients (β), standard errors (SE), and p-values are provided for regressions based on ordinary least squares (OLS) and generalized least squares (GLS). Best-fit lines (based on OLS) are black when GLS regressions were significant (P≤ 0.05) or gray when nonsignificant (P> 0.05).

Using OLS, all three water-stable aggregate size classes were negatively associated with root biomass (0–30 cm depth) (Fig 3). Results for GLS, however, indicated only one marginally significant negative association between water-stable aggregates (0.25–1 mm) and root biomass (Fig 3E) and no appreciable association for other size classes of aggregates. For the soil surface layer (0–10 cm), we found a significant negative association between two size classes of water-stable aggregates (0.25–1 and 1–2 mm) and root biomass (GLS; β = -8.8, SE = 3.8, P = 0.02 and β = -7.8, SE = 3.8, P = 0.04, respectively) and no appreciable association for the largest size class of aggregates (GLS; β = -4.6, SE = 3.5, P = 0.19; results not shown). All three water-stable aggregate size classes were negatively associated with *Carex filifolia* root biomass (0–10 cm depth; Fig 3). All three water-stable aggregate size classes were also negatively associated with log transformed *C*. *filifolia* root biomass (GLS; 0.25–1 mm, β = -0.02, SE = 0.007, P = 0.006; 1–2 mm, β = -0.01, SE = 0.006, P = 0.047; and 2–4 mm β = -0.01, SE = 0.005, P = 0.008).

**Fig 4:**
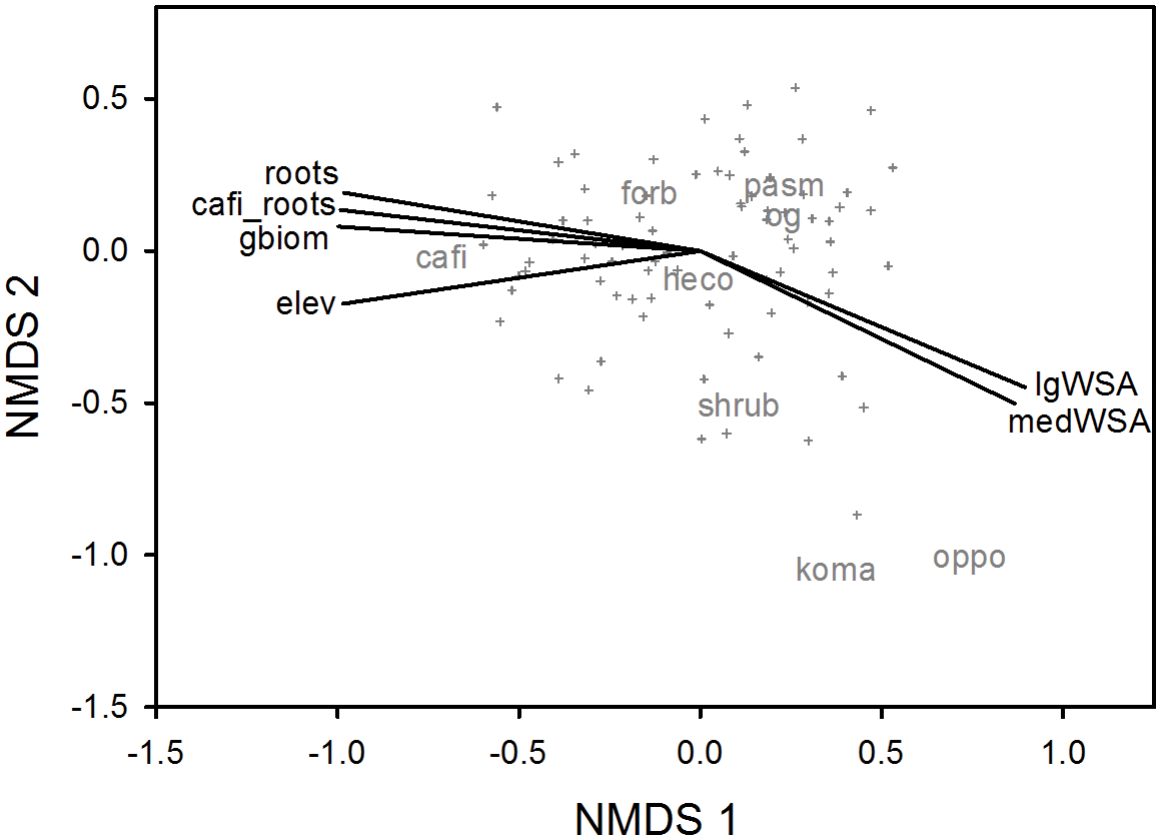
Non-metric multidimensional scaling (NMDS) graph of vegetation sample dissimilarities with best correlating environmental variables plotted as vectors. Sample (i.e. field plot) symbols are crosshairs. Major plant category (e.g. species, functional group) abbreviations in gray include: cafi, *Carex filifolia*; forb, forbs; heco, *Hesperostipa comata*; koma, *Koeleria macrantha*; og, other grasses; oppo, *Opuntia polyacantha*; pasm, *Pascopyrum smithii*; and shrub, *Artemisia* shrubs. Abbreviations for environmental vectors are as follows: cafi_roots, *Carex filifolia* roots (0–10 cm); elev, elevation gain from lowest sampled point; gbiom, dominant graminoid (aboveground) biomass; lgWSA, % water-stable aggregates (2–4 mm [and 0–10 cm soil depth]); medWSA, % water-stable aggregates (1–2 mm); and roots, root biomass (0–30 cm). The stress computed for this ordination was 0.23.

A stable NMDS ordination was found after 20 iterations with a final stress of 0.23 (Fig 4). The NMDS graph illustrates that as distance between plots or species objects increases in the graph, the similarity between them decreases. Species (or functional groups) plotted near the graph’s origin tended to occur in all plots (e.g. *H*. *comata*). The NMDS then indicates gradients in plant composition. Variation in plant community dissimilarity was correlated with several environmental factors (shown as vectors, Fig 4). Elevation gain explained the largest amount of variation in the NMDS graph (R^2^ = 0.52, P< 0.001) followed by total root biomass (R^2^ = 0.23, P< 0.001), % water-stable aggregates (1–2 mm; R^2^ = 0.17, P< 0.001), *Carex filifolia* root biomass (R^2^ = 0.16, P = 0.003), % water-stable aggregates (2–4 mm; R^2^ = 0.13, P = 0.006), and dominant graminoid biomass (R^2^ = 0.13, P = 0.003; Fig 4). The close association between the dominant plant species *C*. *filifolia* and several environmental vectors suggests that upper portions of the gradient (e.g. 0.6 to 1.2 m elevation gain) tended to be dominated by *C*. *filifolia* and had greater total root biomass, *C*. *filifolia* root biomass, and graminoid (aboveground) biomass. This sedge species and relevant vectors were also inversely related to two measures of % water-stable aggregates (and somewhat to the small aggregate size class; R^2^ = 0.06, P = 0.10). These multivariate relationships indicate that positive plant productivity-soil aggregate stability associations were not apparent (e.g. biomass vectors were opposite to aggregate stability vectors) and possibly confounded by the identity of the dominant plant species per sampling point. Specifically, water-stable aggregates (1–2, 2–4 mm) were negatively associated with sampling points dominated by *C*. *filifolia* biomass (foliar and roots) and with prevalent aboveground graminoid and root biomasses.

## Discussion

We found no evidence for a positive association between soil aggregate stability and plant properties. It is important to recall that predicted positive associations may be disrupted by other factors that affect plant productivity (or soil aggregate stability). Contrary to predicted associations, we found several significant and marginally significant negative associations between soil aggregate stability and plant productivity. All regression slope coefficients (β) were negative thereby indicating a tendency for negative associations across all comparisons. Since our study was of a single site, our findings may not be generalizable to other sites or rangeland types. However, our results are not atypical since others have failed to detect positive associations between measures of soil aggregate stability and crop yield [15], plant cover [16, 17], total peak aboveground biomass [18], and root biomass [19, 20]. Our prediction was likely wrong because plant biomass (i.e. dominant graminoid peak aboveground biomass, total peak aboveground biomass) data were nonzero (i.e. all areas were vegetated) and variation in dominant plant taxa may confound the predicted relationship. In other words, patches of highly productive graminoids had lower levels of % water-stable soil aggregates than expected. Here the subtle gradient in elevation across the sampled site (Fig 1) coincided with a shift in plant community composition, especially dominance by *Carex filifolia* (Fig 4). *Carex filifolia* is a native sedge species that provides valuable forage to livestock and wildlife [32]. *Carex filifolia* also produces prolific coarse roots (Fig 4) believed to help stabilize soil ([45] and citations therein). The negative correlation between *C*. *filifolia* root biomass and % water-stable aggregates (Fig 3) is important and perplexing since both variables are believed to be positively related to “soil/site stability” (i.e. land’s capacity to resist erosion). Others have reported that plant species may affect aggregate stability [4, 10, 46].

Sedge species (*Carex* spp.) are often regarded as nonmycorrhizal [47] which may relate to the distinct negative association between *C*. *filifolia* abundance and measures of soil aggregate stability (Figs 3 and 4). However, this and other sedge species associate with arbuscular mycorrhizal fungi (AMF) [31, 48] but may be relatively unresponsive to AMF. Furthermore, the Northern Great Plains is dominated by C_3_ grasses in the Pooideae subfamily which tend to have subtle responses to AMF [49]. Dominant plant taxa in the Northern Great Plains appear much less capable of dramatically affecting soil aggregate stability levels than dominant taxa in the tallgrass prairie region of the Central Plains [50]. We also hypothesize that invasive non-native plants may increase, decrease, or not affect soil aggregate stability. For example, areas heavily invaded by AMF-dependent taxa [46, 51] may have higher levels of aggregate stability than noninvaded grasslands. Thus, variation in dominant plant species, native or invasive, may affect soil aggregate stability and confound predicted associations between aggregate stability and plant properties.

Does a general positive association between plant biomass and soil aggregate stability exist? Mechanistic studies on the formation of water-stable aggregates indicate that aggregate formation is based partly on plant roots and mycorrhizal fungi [3–5, 10]. Furthermore, mensurative experiments with plant biomass (or cover) that include zeros (i.e. samples include some unvegetated areas) tend to report positive associations between soil aggregate stability and plant properties [7–9]. We hypothesize that the probability of detecting a positive association between soil aggregate stability and plant biomass is at least partly dependent on local minimums and whether datasets include plant biomass data with zeros (i.e. nonvegetated areas). Datasets with varying plant biomasses and nonzero data (i.e. all samples were vegetated to varying degrees) often either fail to detect positive associations between soil aggregate stability and plant properties [16–18] or detect negative associations (Figs 2 and 3). The hypothesized importance of local minimums coupled with effects of dominant plant species are likely to affect the reliability of soil aggregate stability as an indicator variable.

## Conclusions

We found new evidence for trade-offs between plant biomass and soil aggregate stability. Dominant plant species seemingly have the capacity to confound predicted positive relationships for soil aggregate stability and grassland productivity. These results complement existing regional studies in the Northern Great Plains which failed to detect a positive association between soil aggregate stability and plant biomass. Variation in plant species composition is likely a main factor obscuring detection of positive associations between soil aggregate stability and plant biomass.
